# Comparison of tonsil-oral-scrubbing with serum, oral fluid, and tonsil scraping to detect PRRSV RNA in sows over time following live virus inoculation

**DOI:** 10.3389/fvets.2024.1506995

**Published:** 2024-11-14

**Authors:** Peng Li, Thomas Petznick, Emily Pratt, Guilherme Cezar, Kelly Will, Mafalda Mil-Homens, Hao Tong, Isadora Machado, Daniel C. A. Moraes, Rodrigo C. Paiva, Alexis Berte, Onyekachukwu H. Osemeke, Paul Yeske, Gustavo S. Silva, Daniel C. L. Linhares

**Affiliations:** ^1^College of Veterinary Medicine, Iowa State University, Ames, IA, United States; ^2^ArkCare, Omaha, NE, United States; ^3^Swine Vet Center, Saint Peter, MN, United States

**Keywords:** PRRSV, detection, sow, TOSC, tonsil scraping, oral fluid, serum, surveillance

## Abstract

**Introduction:**

Current common sample types for sows, such as serum and tonsil scraping, require snaring the animals, which can be labor-intensive and raise concerns regarding animal welfare. Obtaining oral fluids (OF) from individual sows in field conditions presents challenges, as not all sows readily respond to the rope method. The Tonsil-Oral-Scrubbing (TOSc) collector allows for the rapid retrieval of fluids from the sow’s oral and tonsillar areas without the need for snaring. Previous studies have reported comparable detection rates of porcine reproductive and respiratory syndrome virus (PRRSV) RNA between TOSc and tonsil scraping, with significantly higher positivity observed in TOSc compared to serum in acutely infected sows.

**Methods:**

Given that PRRSV RNA detection rates can vary among different sample types and fluctuate over time, this field study aimed to compare PRRSV real-time reverse-transcription polymerase chain reaction (RT-rtPCR) positivity and cycle threshold (Ct) values between TOSc, serum, OF, and tonsil scraping at three time points following live-virus inoculation (LVI) in sows. This study was conducted within a breeding herd attempting to eliminate PRRSV following an outbreak. Four sample types were collected from each of the 61 conveniently selected sows at 30, 60, and 90 days post-LVI in the order of OF, TOSc, tonsil scraping, and serum, and subsequently tested for PRRSV RNA.

**Results:**

The results indicated that TOSc and tonsil scraping exhibited decreased PRRSV RNA detection rates over time, whereas the detection rates for OF and serum remained relatively stable. Moreover, the median Ct values for TOSc and tonsil scraping were numerically lower than those for OF and serum at all sampling points. Specifically, tonsil scraping demonstrated significantly higher PRRSV RNA positivity than the other three sample types. TOSc also exhibited significantly higher PRRSV RNA positivity than OF and serum at both 30 and 60 days post-LVI. By 90 days post-LVI, there was a significant difference in the PRRSV RNA detection rates between TOSc and tonsil scraping. However, no significant difference was observed between TOSc and OF or between TOSc and serum. According to the RT-rtPCR results, most PRRSV RNA-positive sows detected via TOSc and tonsil scraping turned negative by 90 days post-LVI, although a small proportion remained positive. Conversely, a small number of previously negative sows tested positive at 60 and 90 days post-LVI, indicating an intermittent mode of PRRSV RNA detection for both sample types.

## Introduction

One major challenge in managing and eliminating porcine reproductive and respiratory syndrome virus (PRRSV) in breeding herds is viral persistence at the population level, which reflects long-term stability. The American Association of Swine Veterinarians (AASV) defines a breeding herd as achieving PRRSV stability when there is diagnostic evidence of a sustained lack of viremia in pigs at weaning for 13 consecutive weeks (1). A study in 2021 reported a median time to stability (TTS) of 35 weeks, with significant variation ranging from 23 to 49 weeks (2). Commonly used sample types, such as tongue fluids (TF) (3), processing fluids (PF) (4, 5), and family oral fluids (FOF), (6, 7) primarily originate from suckling piglets and may miss PRRSV activity in sows. Given that sows are a major source of PRRSV that can be transmitted to piglets (8), undetected PRRSV in the breeding herd poses a significant challenge to the success of virus management and elimination programs in the field.

Thus, a practical and reliable tool is needed to directly sample the sows. Common sample types for detecting various pathogens in sows include serum and tonsil scraping, (9, 10) with occasional use of oral fluid (OF) (11, 12). These samples can be used to detect PRRSV RNA at different prevalence levels; however, tonsil scraping provides higher herd sensitivity than serum and other sample types for identifying long-term PRRSV carrier pigs, likely due to localized virus genome presence in lymphoid tissues such as the tonsil during this phase of infection (10).

However, both serum and tonsil scraping are time consuming and labor intensive for large-scale screening. Moreover, both methods require restraining the sows, which can cause stress and negatively impact animal welfare. In contrast, oral fluid is a more animal-friendly option often used for population-based sampling (6, 13), although very few reports document its use in individual sows. One study indicated a wide variability in the successful collection rate of OF, ranging from 14.6 to 67.4% (12).

Recently, Peng et al. (14) reported a novel sow sampling tool, TOSc, adapted from a sow collector reported for the test-and-removal of African swine fever virus-infected sows in China (14). TOSc collects biological samples from the oral and tonsillar areas of sows within seconds without requiring snaring and shows comparable PRRSV RNA detection rates to tonsil scraping (14).

Data from an acutely PRRSV-infected farm showed that TOSc samples demonstrated 100% positivity for PRRSV RNA, whereas tonsil scrapings yielded a positivity rate of 73.3%, and serum samples showed only 16.8% positivity in 30 sows. However, this study did not compare OF with TOSc. Moreover, since the PRRSV detection rate varies among different sample types and changes over time (13), it remains unknown whether TOSc exhibits a similar PRRSV detection pattern to other sample types.

Thus, this field study aims to compare TOSc with serum, OF, and tonsil scraping to detect PRRSV RNA in sows at various time points post-whole-herd exposure to a wild-type PRRSV virus in one breeding herd farm.

## Methods

This prospective observational study was conducted following gestating sows after a herd closure and a live virus exposure program to eliminate PRRSV. The breed-to-wean farm was a continuous production system with 4,500 sows located in the Midwestern US. Before the outbreak with a PRRSV 1–4-4 lineage 1C.5 variant, the farm was naïve (status IV as classified by AASV PRRSV status classification) (1). At 30, 60, and 90 days post-LVI, four sample types were collected and tested from each of the 61 conveniently selected parity zero sows housed in the gestation stalls: OF, TOSc, tonsil scraping, and serum were collected consecutively for each sow, tested, and compared for PRRSV RNA detection rate and Ct values. TOSc was performed by the same person throughout the study, but tonsil scraping, serum, and OF were collected by different people. The Institutional Animal Care and Use Committee (IACUC) of Iowa State University approved this study (IACUC-22-101).

### Sample size calculation

The sample size was calculated to be 60 sows, assuming a 15% difference in the PRRSV RNA detection rate at a 70% prevalence, with a 5% alpha level and 80% power.

### OF collection

The OF samples were collected from each sow by placing a rope in front of the gestation stall. In brief, a rope that is 1.59 cm in diameter and made of 100%-cotton was hung in the front of each stall for 30 to 45 minutes. To harvest the oral fluid, the rope was put in a plastic bag and then wrung. Sample with a volume of ≥ 1.0 mL was defined as a successful OF collection (12).

### TOSc collection

TOSc was collected as previously described with some modifications (14). Samples were collected with the sows restrained and mouths held open to decrease the time required to collect all sample types to reduce stress for each pregnant sow as per the farm management’s request. Briefly, the sows were restrained with a snare, and the mouths were held open with a metal mouth gag. The head part of the collector was directed toward the tonsillar area of the mouth and moved back and forth for 10 s. The qualified sample was viscous and mucous-like. The sample was transferred to a 50-ml conical tube (Corning Science Mexico S.A. de C.V., Tamaulipas, Mexico) prefilled with 3 mL of PBS.

### Tonsil scraping

After the TOSc samples were collected, tonsil scraping samples were collected for each sow as previously described (10). When the sow was restrained, a long-handled metal spoon was used to scrape the soft palatine tonsil. The spoon was inserted with the bowl side up, avoiding contact with non-tonsillar tissues. The qualified sample was viscous and mucous-like. Then, the sample on the spoon was transferred using a polyester swab (Puritan Medical Products Company, LLC, Guilford, ME, USA) to a 5 mL conical tube (Corning Science Mexico S.A. de C.V., Tamaulipas, Mexico) containing 1 mL of PBS. Then, the metal spoon was disinfected using a disposable disinfecting wipe (Clorox® Disinfecting Wipes, Clorox Company, US) and dried with a paper towel.

### Serum collection

Precaval-vein blood was also collected using B.D. Vacutainer (Becton Dickinson and Company, Franklin Lakes, NJ, USA) from the snared sows. Blood was allowed to clot by leaving it undisturbed at room temperature for 15–30 min. The serum was then separated from the clot by centrifugation at 1500 g for 5 min.

### Diagnostic testing

All samples were tested at the Iowa State University Veterinary Diagnostic Laboratory for PRRSV RNA using validated commercially available extraction kits (MagMax, Thermo Fisher, Austin, USA) and PRRSV RT-rtPCR kits (VetMAX™ PRRSV EU and NA 3.0 Kit, Austin, USA) per to the manufacturer’s instructions. Test results with a cycle threshold (Ct value) <40 were considered PRRSV RNA-positive.

### Statistical analysis

Descriptive statistics were used to report the PRRSV RNA detection rate, distribution of Ct values for each sample type, and changes in PRRSV status for each sow based on TOSc and tonsil scraping RT-rtPCR results. A logistic mixed regression model was employed to assess the difference in the PRRSV RNA detection rate as a function of different sample types, different collection times, and the interaction between sample types and collection times. Sow ID and the interaction between sow ID and collection times were included as random effects to account for individual variability and time-specific deviations within each sow. An autoregressive covariance structure [AR(1)] was selected for the residuals within each sample type nested in each sow ID by comparing Akaike Information Criterion (AIC) values among different covariance structures. The Tukey–Kramer test was used to compare the *post hoc* pairwise differences in the detection rates between sample types. The Dunn’s test was conducted to assess if there was a difference in the Ct values from positive samples among different sample types. All analyses were performed using the nlme package from R program 4.2.2 (15, 16).

## Results

### Comparison of PRRSV RNA detection rate among sample types at 30-, 60-, and 90-days post-LVI

**Figure 1:**
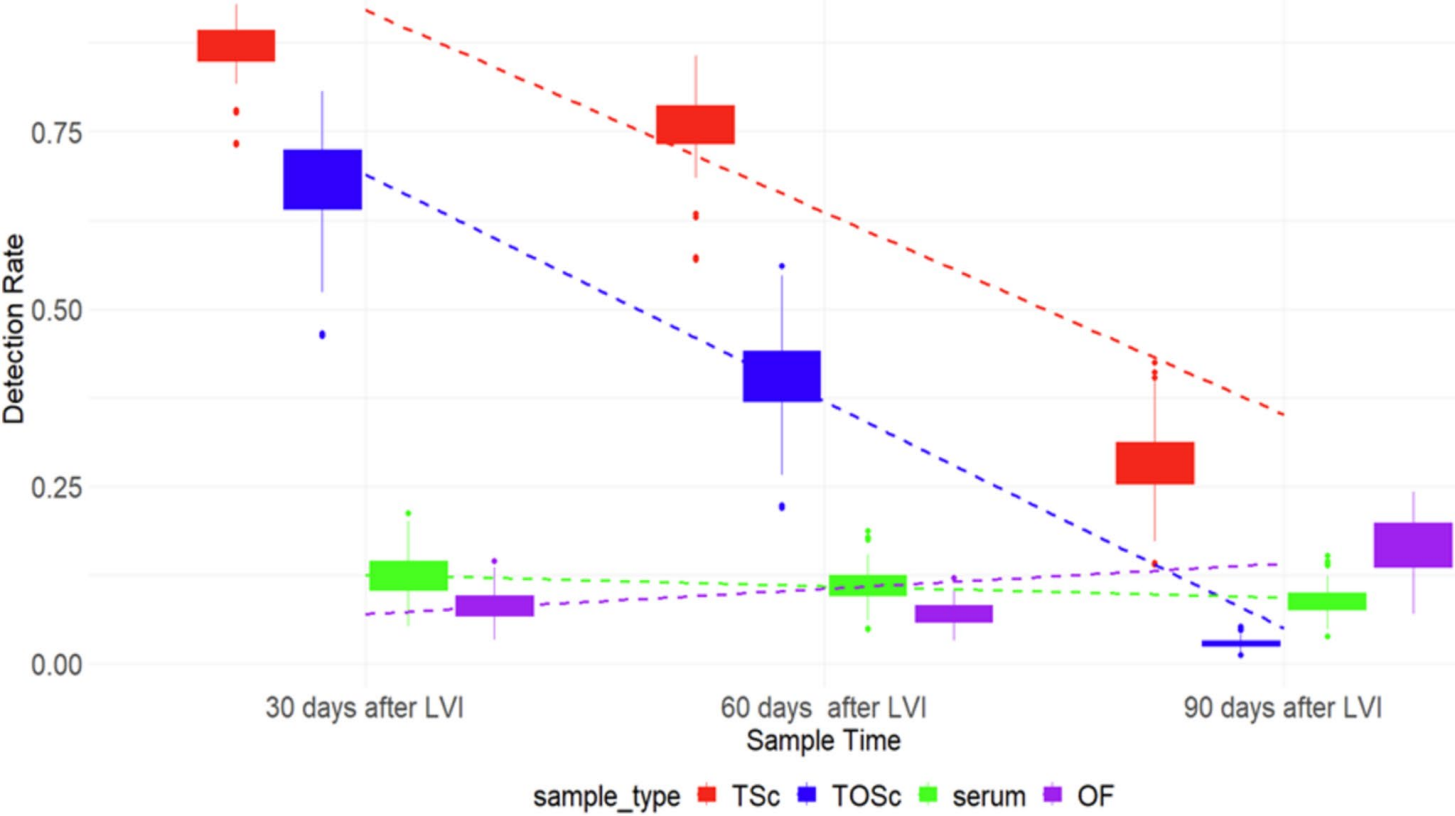
PRRSV RNA detection rate among different sample groups over time. TSc, tonsil scraping, TOSc, tonsil-oral scrubbing, OF, oral fluid. LVI, live virus inoculation.

There was a similar pattern of PRRSV RNA detection rates for TOSc and tonsil scraping, which decreased over time, while that of serum and OF remained relatively flat (Figure 1).

At 30 days post-LVI, tonsil scraping (85.3, 95% CI, 71.2–93.2%) had a significantly higher PRRSV RNA detection rate than TOSc (66.3, 95% CI, 50.9–78.7%) (Tukey’s test, *p* = 0.013), and both tonsil scraping and TOSc had significantly higher detection rates than OF (8.8, 95% CI, 3.3–21.9%) and serum (13.2, 95% CI, 5.9–27.1%) (*p* < 0.001 for all pairwise comparisons). Similarly, at 60 days post-LVI, tonsil scraping (74.2, 95% CI, 62.4–83.3%) showed a significantly higher detection rate than TOSc (40.9, 95% CI, 29.8–53.1%) (*p* < 0.001). Similarly, both tonsil scraping and TOSc showed significantly higher detection rates than OF (8.0, 95% CI, 3.0–19.5%) and serum (12.1, 95% CI, 6.2–22.4%) (*p* < 0.001 for all pairwise comparisons). At 90 days post-LVI, there was a significant difference between TOSc (3.28, 95% CI, 0.5–16.9%) and tonsil scraping (29.5, 95% CI, 17.2–45.7%) (*p* < 0.001), and no significant difference between TOSc and OF (17.86, 95% CI, 5.9–42.6%) or serum (9.84, 95% CI, 3.6–24.1%) (*p* = 0.637, *p* = 0.149) for pairwise comparisons between TOSc and serum and between TOSc and OF, respectively (Table 1 and Figure 1).

**Table 1:** PRRSV RT-rtPCR detection rate among sample types at 30, 60, and 90 days post live virus inoculation.

Sample types	PRRSV detection rate (95% CI)		
	30 days post-LVI	60 days post-LVI	90 days post-LVI
Tonsil scraping	85.3% (71.2–93.2%)^a^	74.2% (62.4–83.3%)^a^	29.5% (17.2–45.7%)^a^
TOSc	66.3% (50.9–78.7%)^b^	40.9% (29.8–53.1%)^b^	3.28% (0.5–16.9%)^b^
Serum	13.2% (5.9–27.1%)^c^	12.1% (6.2–22.4%) ^c^	9.84% (3.6–24.1%)^b^
OF	8.8% (3.3–21.9%)^c^	8.0% (3.0–19.5%)^c^	17.86% (5.9–42.6%)^ab^

^a,b,c^Different letters indicate significant differences in the least-square means of PRRSV detection rate among different sample types at each sampling time point (Tukey test, *p* < 0.05). TOSc, tonsil-oral scrubbing, OF, oral fluid. LVI, live virus inoculation.

### Comparison of PRRSV RT-rtPCR Ct values from positive samples among sample types at 30, 60, and 90 days post-LVI

**Figure 2:**
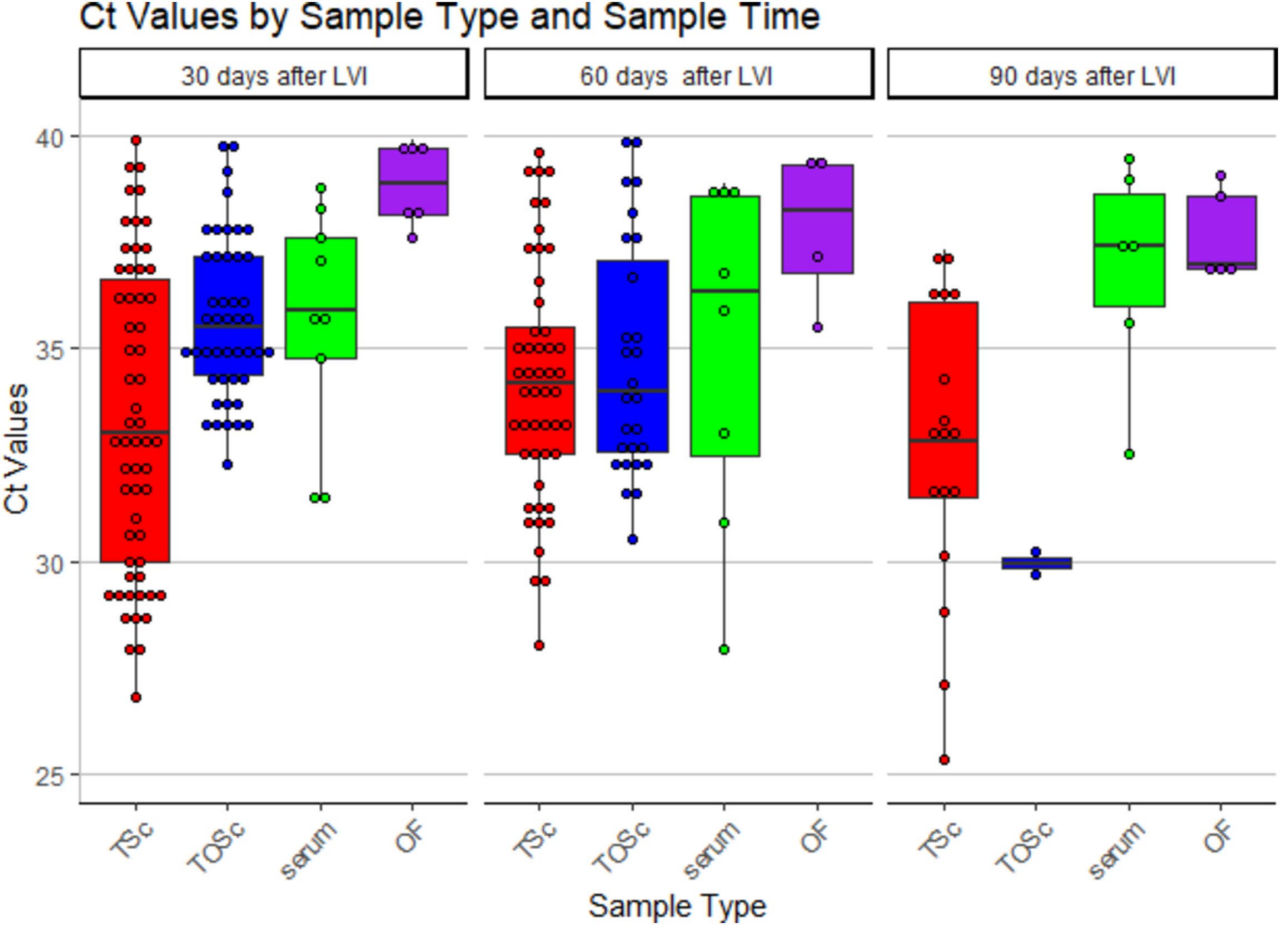
Distribution of PRRSV RNA RT-rtPCR-positive Ct values among different sample groups over time. TSc, tonsil scraping, TOSc, tonsil-oral scrubbing, OF, oral fluid. LVI, live virus inoculation.

Numerically, tonsil scraping and TOSc showed lower median Ct values than OF and serum in RT-rtPCR-positive samples at all sampling points (Table 2 and Figure 2).

**Table 2:** Ct value median and range of PRRSV RT-rtPCR-positive samples among sample types at 30, 60, and 90 days post live virus inoculation.

Ct value median (range)	30 days post-LVI	60 days post-LVI	90 days post-LVI
Tonsil scraping	33.3 (26.8–39.9)^a^	34. 7 (28–39.6)^a^	32.7 (25.3–37.1)^a^
TOSc	35.5 (32.3–39.8)^b^	33.9 (30.5–39.8)^a^	29.9 (29.7–30.2)^a^
Serum	35.7 (31.4–38.8)^ab^	36.0 (30.9–38.9)^a^	36.9 (32.5–39.5)^b^
Oral fluid	38.9 (37.6–39.9)^c^	37.9 (35.5–39.5)^a^	37.7 (36.8–39.1)^b^

^a,b,c^Different letters indicate significant differences in least-square means of Ct values among different sample types at each sampling time point (Dunn’s test). LVI, live virus inoculation.

At 30 days post-LVI, tonsil scraping showed significantly lower median Ct values (33.3, range 26.8–39.9) compared to TOSc (35.5, range 32.3–39.8, Dunn’s Test, *p* < 0.01) and OF (38.9, range 37.6–39.9, *p* < 0.001). TOSc also showed significantly lower median Ct values than oral fluid (*p* = 0.014). At 60 days post-LVI, there was no significant difference between any sample types regarding Ct values (*p* > 0.05). At 90 days post-LVI, the median Ct values of tonsil scraping [32.7, range (25.3–37.1)] and TOSc [29.9, range (29.7–30.2)] were significantly lower (*p* < 0.01 for all pairwise comparisons) than oral fluid [36.9, range (32.5–39.5)] and serum [36.9, range (32.5–39.5)], while there was no significant difference between tonsil scraping and TOSc or between OF and serum (*p* > 0.05).

### PRRSV RNA positive/negative status changes in individual sows over time based on tonsil scraping and TOSc RT-rtPCR results

Based on PRRSV RNA RT-rtPCR results of tonsil scraping (Table 3), from 30 days post-LVI to 60 days post-LVI, 63.9% (39/61) positive sows remained positive, while 21.3% (13/61) positive sows turned negative. There were 6.6% (4/61) negative sows remaining negative; however, 7.9% (5/61) negative sows turned positive.

**Table 3:** PRRSV RNA positive/negative status changes in individual sows over time based on tonsil scraping and TOSc RT-rtPCR results.

Change of tonsil scraping PRRSV RNA status	30 days→60 days	60 days→90 days	30 days→90 days
Pos→pos proportion	63.9% (39/61)	19.7% (12/61)	23.0% (14/61)
Pos→neg proportion	21.3% (13/61)	52.5% (32/61)	62.3% (38/61)
Neg→neg proportion	6.6% (4/61)	19.7% (12/61)	9.8% (6/61)
Neg→pos proportion	7.9% (5/61)	8.2% (5/61)	4.9% (3/61)
Total	100%	100%	100%

Change of TOSc PRRSV RNA status	30 days→60 days	60 days→90 days	30 days→90 days
Pos→pos proportion	26.2% (16/61)	3.3% (2/61)	0
Pos→neg proportion	39.3% (24/61)	36.1% (22/61)	65.6% (40/61)
Neg→neg proportion	13.1% (8/61)	60.7% (37/61)	31.1% (19/61)
Neg→pos proportion	21.3% (13/61)	0% (0/61)	3.3% (2/61)
Total	100%	100%	100%

Pos → pos proportion: proportion of sows that were RT-rtPCR positive at both sampling points. Pos → neg proportion: proportion of sows that were RT-rtPCR positive at the first sampling point and turned negative at the following (60 or 90 days post-LVI) sampling point. Neg → neg proportion: proportion of sows which were RT-rtPCR negative at both sampling points. Neg → pos proportion: proportion of sows which were RT-rtPCR negative at the first sampling point and turned positive at the following (60 or 90 days post-LVI) sampling point.

From 60 days post-LVI to 90 days post-LVI, 19.7% (12/61) positive sows remained positive, while 52.5% (32/61) positive sows turned negative. Conversely, 19.7% (12/61) of sows tested negative at both 60 and 90 days, while 8.2% (5/61) sows tested negative at 60 days and turned positive at 90 days.

When comparing 30 days post-LVI to 90 days post-LVI, 23.0% (14/61) positive sows remained positive, while 62.3% (38/61) positive sows turned negative. A total of 9.8% (6/61) negative sows remained negative, while 4.9% (3/61) negative sows turned positive.

Based on RT-rtPCR results of TOSc (Table 3), from 30 days post-LVI to 60 days post-LVI, 26.2% (16/61) positive sows remained positive, while 39.3% (24/61) positive sows turned negative. There were 13.1% (8/61) negative sows remaining negative; however, 21.3% (13/61) negative sows turned positive.

From 60 days post-LVI to 90 days post-LVI, 3.3% (2/61) positive sows remained positive, while 36.1% (22/61) positive sows turned negative. There were 60.7% (37/61) negative sows remaining negative, while no negative sows turned positive.

When comparing 30 days post-LVI to 90 days post-LVI, no positive sows remained positive, and 65.6% (40/61) positive sows turned negative. A total of 31.1% (19/61) negative sows remained negative, while 3.3% (2/61) negative sows turned positive.

## Discussion

PRRSV RNA detection in different sample types varies and changes at different infection stages (10, 13). Thus, it is important to compare TOSc with other common sample types in terms of PRRSV RNA detection mode over time after live virus exposure. In general, TOSc exhibited a similar PRRSV RNA detection pattern with tonsil scraping but a different pattern than individual OF and serum over time after live virus exposure. While TOSc and tonsil scraping showed a decreased PRRSV RNA detection rate over time, detection rates in OF and serum remained relatively the same. The median Ct values of TOSc and tonsil scraping were numerically lower than OF and serum at each sampling point, indicating the detection of higher quantities of PRRSV RNA in TOSc and tonsil scraping samples.

Specifically, tonsil scraping exhibited a significantly higher PRRSV RNA detection rate than other sample types, including TOSc, at all sampling points except at 90 days post-LVI, where no significant difference in detection rates was observed between tonsil scraping and OF. This finding contrasts with our previous study, which indicated that TOSc had a numerically higher, though not statistically significant, PRRSV RNA detection rate compared to tonsil scraping in 30 acutely infected sows (14).

The discrepancy may be attributed to the fact that, in the current study, TOSc samples were collected while the sows were restrained, with their mouths held open to minimize the time required for sample collection and reduce stress, as requested by farm management. In contrast, the previous study involved collecting TOSc from non-snarred sows, allowing them to chew on the TOSc collector, which likely increased contact between the collector and the tonsillar area compared to when the sows were restrained.

Additionally, in another study (data not published), we collected TOSc from the same 61 sows as in the current study without snaring them approximately 90 days post-LVI. The results showed that 13 out of 61 sows tested positive for PRRSV RNA in TOSc samples (21.3, 10.4–34.2%), compared to only two sows testing positive when they were snared (3.28, 0.5–16.9%) in the present study. This supports previous findings that “not snared” sows yielded numerically higher PRRSV RNA detection rates, significantly lower median Ct values, and significantly higher sample volumes than “snared” sows (17).

TOSc showed a significantly higher detection rate than OF and serum at 30 and 60 days post-LVI. This was within expectation because TOSc can collect biological materials from the tonsillar area, which has visually opaque cell deposits, as examined by histopathology and cytology after placement at room temperature for 5 min (17). Tonsil samples showed extended PRRSV RNA detection up to 251 days after live virus inoculation (10). In contrast, PRRSV RNA was reported to be detected in OF and serum of sows from 8 weeks up to 12 weeks post-live virus exposure (18–20). The observed lack of significant difference in PRRSV RNA detection rates between OF, tonsil scraping, and TOSc at 90 days post-LVI might be due to the poor collection rate of oral fluid as an individual sample type: only 26 OF samples were successfully collected from 61 sows. Similarly, 45 OF were successfully collected from 61 sows at 60 days post-LVI. This decreased the sample size of OF for a valid comparison. Moreover, OF is frequently used as a population-based sample type and was collected from a group of animals rather than from individual animals (6, 21). This was consistent with a previous report documenting various success rates of OF collection in individual adult pigs (12). The ease of collection and similar PRRSV RNA detection pattern with tonsil scraping suggest a comparative advantage of TOSc as an individual sample type to PRRSV RNA detection over time in the sow population compared with other samples.

Based on PRRSV RT-rtPCR results from tonsil scraping samples, 63.9% of positive sows remained positive at 60 days post-LVI. However, 52.5% of positive sows turned negative at 90 days post-LVI, showing an abrupt change of PRRSV RNA positive/negative status in sows at this time point. A similar trend of sow PRRSV RNA positive/negative status change was also observed based on RT-rtPCR results from TOSc, when 39.3 and 36.1% positive sows turned negative at 60 days post-LVI and 90 days post-LVI, respectively. Even 90 days post-LVI, 27.9% of sows were PRRSV RNA positive on tonsil scraping. This was consistent with previous reports showing that PRRSV can persist in tonsils for up to 251 days (10, 22).

Of the sows that tested positive at 90 days post-LVI, some were tested negative at 30 days post-LVI and/or 60 days post-LVI on both sample types. This suggests an intermittent mode of PRRSV RNA detection in sows several months after live virus exposure, similar to what had been described in PRRSV RNA detection in tonsil samples from growing pigs (10, 22), and highlights a need for a continuous surveillance program on sows to better understand the PRRSV dynamics in this subpopulation.

One limitation of the study was that the sampled population were all pregnant gilts (parity 0) and might not reflect the PRRSV dynamics in other parties.

## Conclusion

Taken together, this study demonstrates that TOSc exhibits a detection pattern for PRRSV RNA similar to that of tonsil scraping, indicating its potential usefulness as a proxy for tonsil scraping in PRRSV surveillance among sow populations. Further studies are required to establish the “best practice” for TOSc collection to enhance PRRSV RNA detection rates at various stages of infection.

## Data Availability

The raw data supporting the conclusions of this article will be made available by the authors without undue reservation.

## References

[1] Holtkamp DJ. Proposed modifications to porcine reproductive and respiratory syndrome virus herd classification. J Swine Health Prod. (2021) 29:261–70. doi: 10.54846/jshap/1218

[2] Linhares DCL Trevisan G, Rodger Main DH Linhares L Corzo C, Swine disease management information program, 20–109. (2022). Pork Check Off Research Report, NPPC Final Report Format. Available at: https://porkcheckoff.org/.

[3] Machado IF Magalhães ES Poeta Silva APS Moraes DCA Cezar G Mil-Homens MP. Porcine reproductive and respiratory syndrome virus RNA detection in tongue tips from dead animals. Front Vet Sci. (2022) 9:993442. doi: 10.3389/fvets.2022.993442, PMID: 36213411 PMC9533096

[4] Almeida MN Zhang M Lopez WAL Vilalta C Sanhueza J Corzo CA. A comparison of three sampling approaches for detecting PRRSV in suckling piglets. Prev Vet Med. (2021) 194:105427. doi: 10.1016/j.prevetmed.2021.105427, PMID: 34271476

[5] López WA Zimmerman JJ Gauger PC Harmon KM Bradner L Zhang M. Practical aspects of PRRSV RNA detection in processing fluids collected in commercial swine farms. Prev Vet Med. (2020) 180:105021. doi: 10.1016/j.prevetmed.2020.105021, PMID: 32428814

[6] Almeida MN Rotto H Schneider P Robb C Zimmerman JJ Holtkamp DJ. Collecting oral fluid samples from due-to-wean litters. Prev Vet Med. (2020) 174:104810. doi: 10.1016/j.prevetmed.2019.104810, PMID: 31756669

[7] Almeida MN Zhang M Zimmerman JJ Holtkamp DJ Linhares DCL. Finding PRRSV in sow herds: family oral fluids vs. serum samples from due-to-wean pigs. Prev Vet Med. (2021) 193:105397. doi: 10.1016/j.prevetmed.2021.105397, PMID: 34147958

[8] Pileri E Mateu E. Review on the transmission porcine reproductive and respiratory syndrome virus between pigs and farms and impact on vaccination. Vet Res. (2016) 47:108. doi: 10.1186/s13567-016-0391-4, PMID: 27793195 PMC5086057

[9] Nielsen EO Lauritsen KT Friis NF Enøe C Hagedorn-Olsen T Jungersen G. Use of a novel serum ELISA method and the tonsil-carrier state for evaluation of *Mycoplasma hyosynoviae* distributions in pig herds with or without clinical arthritis. Vet Microbiol. (2005) 111:41–50. doi: 10.1016/j.vetmic.2005.08.009 16171955

[10] Wills RW Doster AR Galeota JA Sur JH Osorio FA. Duration of infection and proportion of pigs persistently infected with porcine reproductive and respiratory syndrome virus. J Clin Microbiol. (2003) 41:58–62. doi: 10.1128/JCM.41.1.58-62.2003 12517825 PMC149563

[11] Pol F Dorenlor V Eono F Eudier S Eveno E Liégard-Vanhecke D. Individual and pen-based oral fluid sampling: a welfare-friendly sampling method for group-housed gestating sows. Prev Vet Med. (2017) 147:58–65. doi: 10.1016/j.prevetmed.2017.08.011, PMID: 29254728

[12] Brent Pepin FL Main R Ramerez A Zimmerman J. Collection of oral fluid from individually housed sows. J Swine Health Prod. (2014) 23:35–7. doi: 10.54846/jshap/854

[13] Henao-Diaz A Ji J Giménez-Lirola L Baum DH Zimmerman J. Understanding and interpreting PRRSV diagnostics in the context of "disease transition stages.". Res Vet Sci. (2020) 131:173–6. doi: 10.1016/j.rvsc.2020.04.023, PMID: 32388019

[14] Li P Silva APSP Moraes DCA Yeske P Osemeke OH Magalhães ES. Comparison of a novel rapid sampling method to serum and tonsil scraping to detect PRRSV in acutely infected sows. Prev Vet Med. (2024) 223:106082. doi: 10.1016/j.prevetmed.2023.106082, PMID: 38176150

[15] R Core Team. R: A language and environment for statistical computing. Vienna: R Foundation for Statistical Computing Vienna (2019).

[16] Zhang H Lu N Feng C Thurston SW Xia Y Zhu L. On fitting generalized linear mixed-effects models for binary responses using different statistical packages. Stat Med. (2011) 30:2562–72. doi: 10.1002/sim.4265, PMID: 21671252 PMC3175267

[17] Li P Silva APP Tong H Yeske P Dalquist L Kelly J. Characterizing best practices for tonsil-oral-scrubbing (TOSc) collection for PRRSV RNA detection in sows. Porcine Health Manag. (2024) 10:37. doi: 10.1186/s40813-024-00385-7, PMID: 39375800 PMC11457483

[18] Trang NT Hirai T Yamamoto T Matsuda M Okumura N Giang NTH. Detection of porcine reproductive and respiratory syndrome virus in oral fluid from naturally infected pigs in a breeding herd. J Vet Sci. (2014) 15:361–7. doi: 10.4142/jvs.2014.15.3.361, PMID: 24690609 PMC4178137

[19] Woonwong Y Kedkovid R Arunorat J Sirisereewan C Nedumpun T Poonsuk K. Oral fluid samples used for PRRSV acclimatization program and sow performance monitoring in endemic PRRS-positive farms. Trop Anim Health Prod. (2018) 50:291–8. doi: 10.1007/s11250-017-1428-z, PMID: 28980168

[20] Batista L Dee SA Rossow KD Deen J Pijoan C. Assessing the duration of persistence and shedding of porcine reproductive and respiratory syndrome virus in a large population of breeding-age gilts. Can J Vet Res. (2002) 66:196–200. PMID: 12146892 PMC227004

[21] Hernandez-Garcia J Robben N Magnée D Eley T Dennis I Kayes SM. The use of oral fluids to monitor key pathogens in porcine respiratory disease complex. Porcine Health Manag. (2017) 3:7. doi: 10.1186/s40813-017-0055-4, PMID: 28405463 PMC5382517

[22] Linhares DC Cano JP Wetzell T Nerem J Torremorell M Dee SA. Effect of modified-live porcine reproductive and respiratory syndrome virus (PRRSv) vaccine on the shedding of wild-type virus from an infected population of growing pigs. Vaccine. (2012) 30:407–13. doi: 10.1016/j.vaccine.2011.10.075, PMID: 22063389

